# Efficacy of transcutaneous electrical nerve stimulation in people with pain after spinal cord injury: a meta-analysis

**DOI:** 10.1038/s41393-022-00776-z

**Published:** 2022-03-11

**Authors:** Ye Yang, Yun Tang, Huiqing Qin, Jianwen Xu

**Affiliations:** Department of Rehabilitation Medicine, The First Affiliated Hospital of Guangxi Medical University, Nanning, Guangxi China

**Keywords:** Rehabilitation, Outcomes research

## Abstract

**Study Design:**

Meta-analysis.

**Objectives:**

This study aimed to evaluate the effect of transcutaneous electrical nerve stimulation in people with pain after spinal cord injury by meta-analysis.

**Methods:**

Reviewed PubMed, Embase, Cochrane library, as well as China National Knowledge Infrastructure (CNKI), Wanfang, and Vip databases to search the randomized controlled trials of pain after spinal cord injury through transcutaneous electrical nerve stimulation from the beginning of the library to March 2021, and analyze the literature with RevMan 5.3 software and the bias in the literature with STATA 12.0 software.

**Results:**

There are six randomized controlled trials in the study with 165 cases. 83 cases in the test group were given transcutaneous electrical nerve stimulation, and 82 cases in the control group used sham stimulation or other treatments. Meta-analysis results showed the experimental group’s visual analog scale (MD = −1.52, 95%CI, −2.44 to −0.60, *P* = 0.001) and short-form McGill pain questionnaire scores (MD = −0.70, 95% CI, −1.03 to −0.25, *P* = 0.002) were lower than those of the control group.

**Conclusions:**

Transcutaneous electrical nerve stimulation has some clinical therapeutic effects on persons with pain after spinal cord injury, but due to the lack of literature, the sample size is not large, and clinical trials need to be further improved later.

## Introduction

Pain is one of the most common complications after spinal cord injury (SCI). That is, approximately 53%–80% of patients experience different types of pain after SCI [1]. Pain is defined as an unpleasant sensory and emotional experience associated with actual or potential tissue damage or described in terms of such damage [2]. According to the International Spinal Cord Injury Pain (ISCIP) Classification, pain after SCI is mainly classified as nociceptive pain (musculoskeletal or visceral) and neuropathic pain (at-level or below-level) [3]. The different types of pain have varying clinical characteristics. Musculoskeletal pain is usually described as dull, aching, movement-related, and can be relieved by rest. Visceral pain is frequently located in the abdominal region with preserved innervation and is dull and cramping. Neuropathic pain is commonly described as sharp, shooting, burning, or electrical. Generally, abnormal sensory responsiveness (hyperesthesia or hyperalgesia) is observed [4]. Raichle et al. [5] not only assessed the physical functions of 157 individuals with chronic pain after SCI, but also several psychosocial variables including coping, catastrophizing, pain-related beliefs, and social support, which advised the etiology of post-SCI pain may be correlated with multiple factors.

Transcutaneous electrical nerve stimulation (TENS) is a non-invasive, inexpensive, safe, and easy-to-use electrical method that delivers specific electrical pulses via the skin to the body to stimulate nerves to relieve pain and treat disease [6]. This modality effectively increases blood circulation in the active area and improves pain [7, 8]. Clinically, it can be used to treat different diseases based on stimulation frequency, intensity, and electrode placement. Stimulation frequency is classified as high (>50 Hz) and low (<10 Hz). Intensity is determined based on individual response, either sensory- or motor-level TENS [9]. Electrodes were commonly placed in opposite directions or juxtaposition. In clinical treatments for pain, low-frequency stimulation with electrodes placed near the affected area is typically applied [10]. TENS has been widely used to improve pain and it was found the importance of appropriate stimulation parameters in enhancing analgesia [11]. Several clinical studies have reported that appropriate stimulation parameters are important to relieve central (cerebrovascular accidents and SCI) [12, 13] and peripheral (diabetic peripheral neuropathy and cancer) [14, 15] pain. However, recent studies have shown that its long-term effects are inconsistent [16, 17]. Recently, systematic reviews and meta-analyses have investigated the role of TENS in the treatment of postpartum pain, post-multiple sclerosis pain, and trigeminal neuralgia [18–20]. However, there is no study has reported the efficacy of TENS among people with post-SCI pain. Therefore, this study used the available evidence collected and integrated from relevant clinical trials to assess the efficacy of TENS against pain after SCI.

## Methods

### Literature Search

**Fig. 1:**
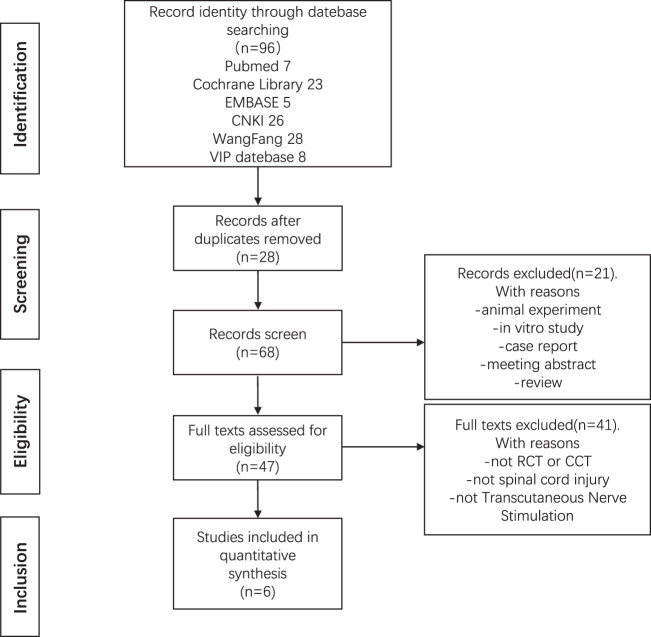
The process of the literature search.

Two researchers searched PubMed (1966 to March 2021), Embase (1974 to March 2021), Cochrane Library (2005 issue 4), China National Knowledge Infrastructure (CNKI) (1999 to March 2021), Wanfang Digital Journal Full Text Database (1998 to March 2021), Vip Chinese Science and Technology Journal Database (VIP) (1989 to March 2021). Chinese search terms are “spinal cord injury”, “spinal fracture”, “pain”, “Transcutaneous Electrical Nerve Stimulation”, “electrical nerve stimulation”, “TENS”, “random”, English search terms are “Spinal Cord Transection”, “Posts Myelopathy”, “Spinal Cord Contusion”, “Pain”, “ache”, “Transcutaneous Electric Nerve Stimulation”, “Percutaneous Electric Nerve Stimulation”, “TENS”, “Transcutaneous Electric Stimulation”, “Transdermal Electrostimulation”, “Transcutaneous Electrical Nerve Stimulation”, “Percutaneous Neuromodulation Therap*”, “Percutaneous Electrical Neuromodulation*”, “Analgesic Cutaneous Electrostimulation”, “Electroanalgesia*”. We combined the subject word with a free word to acquire better retrieval results. Take the PubMed database as an example, and the search strategy is shown in Fig. 1.

### Inclusion criteria

(1) Randomized control trial (RCT) (2) the article is written in Chinese or English (3) whether the blind method is used. (4) meeting the diagnostic criteria for spinal cord injury (International standards for neurological classification of spinal cord injury (revised 2019)) and the patient presents with painful symptoms (5) the intervention uses TENS for the experimental group, and the control group uses sham stimulation or other therapeutic measures (6) evaluation indexes were: Visual analog scale (VAS), short-form McGill pain questionnaire (SF-MPQ), Brief Pain Inventory (BPI), neuropathic pain scale (NPS).

### Exclusion criteria

(1) Basic experiments; (2) Case reports; (3) Summary of meetings; (4) Overview; (5) Full text is not available; (6) Unpublished or duplicate literature.

### Literature extraction

The literature was extracted independently by two researchers (YY and QHQ). The title and abstract were read first for screening, incompatible literature was eliminated, and then the full text was read to screen the literature according to the inclusion and exclusion criteria. Under the circumstance of some differences, it would be resolved by discussions between the 2 investigators. If still unsolvable, the final judgment will submit to third investigators (YT).

### Document quality evaluation

The quality of the literature was assessed according to the RevMan 5.3 software provided by the Cochrane Collaboration Network (https://training.cochrane.org/online-learning/core-software-cochrane-reviews/revman). All included studies were classified into three levels: (1) how random sequences generate (2) allocation concealment (3) application of blind (4) inadequate outcome data (5) whether selecting to report outcome (6) other possible bias. According to The Cochrane Collaboration Handbook [21], the included studies were divided into three risk categories: low, uncertain, and high.

### Outcome indicators

VAS, SF-MPQ, BPI, NPS were used to score pain. Among them, SF-MPQ includes the Pain rating index (PRI) (which in turn contains 11 sensory categories: PRI-sensory and 4 affective categories: PRI-affective, Total pain rating index score: PRI-total), present pain index (PPI), Number of words chosen (NWC), and VAS. VAS is the most commonly used pain-related scale, SF-MPQ can score the individual’s feelings and emotions during pain. VAS and SF-MPQ were mainly used to assess pain in the included studies.

### Statistical methods

Meta-analysis was conducted using RevMan 5.3 software. Heterogeneity analysis was performed first. If I^2^ < 50%, it is considered that no heterogeneity or small heterogeneity among studies, the fixed-effects model could be used to combine effect sizes. If I^2^ > 50%, it is thought that there is more significant heterogeneity among the studies, and the random-effects model was selected to combine effect sizes. Sensitivity analysis to determine the source of heterogeneity. Since continuous outcomes measurements were used, the mean difference (MD) was used to analyze the data effect indicator, and 95% confidence intervals (CI) indicated the effect size. And *P* < 0.05 was considered statistically significant. Funnel plots were drawn with STATA 12.0 software to reveal the presence of publication bias.

## Results

### The literature search results

After the search, 96 papers were initially obtained, and the 28 duplicate documents were removed by Endnote software. After reading the title and abstract, we removed the reviews of eight articles, 10 basic experiments, one case report, two abstracts of the meeting, a total of 21 pieces of literature. After reading the complete text, four full texts could not be obtained, eight trials were not RCT, and 29 studies did not meet the inclusion criteria. Finally, the six papers were included for Meta-analysis. The literature search process is shown in Fig. 1.

### Basic features of literature

A total of 165 subjects were included in the six studies. The test groups all received TENS, but in the control group, three studies [22–24] used sham stimulation (electrode placement without corresponding stimulation), the rest used visual illusion (VI), fluoxetine, and magneto-thermo-vibration therapy (a kind of electrotherapy), respectively. 83 persons were in the test group, and 82 were in the control group. The VAS was used as the final evaluation index in all six treatments. Three studies used the SF-MPQ for evaluation, Çağla, Ö [25] also used the BPI and NPS index as outcome observation, and Xiao-Hong W [26] used the Beck Depression Self-Rating Scale to assess the psychological situation of people after pain. The basic characteristics of the included literature are shown in Table 1.

**Table 1:** The main characteristics and the quality assessment results of the included studies.

Author Year	Gender	Age of patient	Kinds of SCI	Sample size		Intervention	The protocol of TENS	How to place the electrodes	Course of treatment	Outcome
	Male/Female		Complete/ Incomplete		study/control					
Celik, E.C 2013	24/9	36.55 ± 10.36	23/10		17/16	TENS/sham TENS	pulse frequency 4 Hz, pulse duration 200 ms, and pulse amplitude 50 mA	the proximal and two to the distal parts of the region with pain	30 min a day for 10 days	VAS
Vitalii, C 2014	19/2	30.38 ± 6.91	4/17		11/10	TENS/sham TENS	pulse frequency 4 Hz, pulse duration 200 ms, and pulse amplitude 50 mA	the proximal and two to the distal parts of the region with pain	30 min a day For 10 days	VAS
Xia B 2015	32/16	34.55 ± 8.72	33/19		26/26	TENS/sham TENS	Pulse frequency, 2 Hz; pulse duration, < 200 ms; and pulse amplitude, 50 mA	The region with pain.	20 min, three times a week for 12 weeks	VAS SF-MPQ
Çağla, Ö 2015	18/6	32.33 ± 12.97	17/5		12/12	TENS/VI	pulse frequency 80 Hz, the pulse duration 180 µs	The region with pain.	30 min a day, 5 days per week for 2 weeks.	VAS NPS BPI
Xiao-hong W 2011	8/3	36.45 ± 6.55	3/8		5/6	TENS/fluoxetine	Pulse frequency 90 Hz; pulse duration 100 ms; pulse amplitude 40 mA	The region with pain.	20 min, twice a day for 4 weeks	SF-MPQ BDI
Xue-qiang W 2009	22/14	36.55 ± 10.36	14/22		12/12	TENS/Magneto Thermo-vibration therapy	First week Pulse frequency 2 Hz, pulse duration <0.2 ms;	The region with pain.	30 min a day, for 2 weeks	SF-MPQ

### Document quality assessment results

Three [22, 23, 27] did not specifically describe the randomization method in the six randomized controlled studies. Only Xia B [24] mentioned the use of allocation concealment and long-term follow-up, none of the six studies mentioned whether the blinding method was used, and three cases of shedding were described in three studies [24–26]. The document quality Jadad score results are shown in Table 2, and the evaluation results using the Cochrane Bias Risk Assessment Tool are shown in Supplementary Fig. 2.

**Table 2:** Quality of the literature.

Author Year	Random sequence generation	Allocation concealment	Blinding	Follow-up Visit	Complete Outcome data	Modified Jadad score
Çağla, Ö. 2015	Random-number sequence	Not mentioned	Unclear	Not mentioned	Yes	4
Celik, E.C 2013	Not mentioned	Not mentioned	Not mentioned	Not mentioned	Not mentioned	2
Vitalii, C 2014	Not mentioned	Not mentioned	Not mentioned	Not mentioned	Not mentioned	2
Xia B 2015	computer-generated random-number sequence	Yes	Not mentioned	Yes	Yes	5
Xiao-hong W 2011	random-number sequence	Not mentioned	Not mentioned	Not mentioned	Yes	4
Xue-qiang W 2009	Not mentioned	Not mentioned	Not mentioned	Not mentioned	Not mentioned	2

### Meta-analysis results

#### Changes in VAS scores

**Fig. 2:**
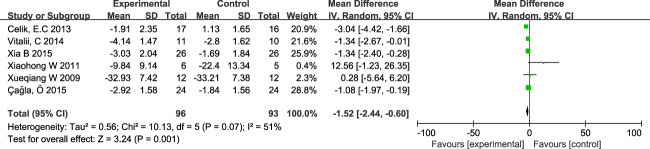
Forest plot of VAS. Compared with control groups, VAS scores in TENS groups were decreased. (‘‘mean’’ represent the mean of VAS for each study, ‘‘total’’ represents how many patients were involved in the study, ‘‘weighted’’ represents the weight of each study in the meta-analysis.).

In the six included studies, 165 subjects all used VAS as an outcome observation indicator. Because of the different values used in the literature to quantify pain (Two studies scored out of 100 on the VAS scale and the other studies scored out of 10), the difference between pre-and post-treatment was used as the result of the analysis. The results show that heterogeneity is significant (I^2^ = 51%, *P* = 0.07), there was a small heterogeneity among studies, and the random effect model was being used. As shown in Fig. 2, the difference between the observation and control groups was statistically significant (the mean difference was −1.52, 95%CI, −2.44 to −0.60, *P* = 0.001), and it can be concluded that a decrease in VAS scores after TENS in individuals with pain after SCI.

#### Changes in SF-MPQ

**Fig. 3:**
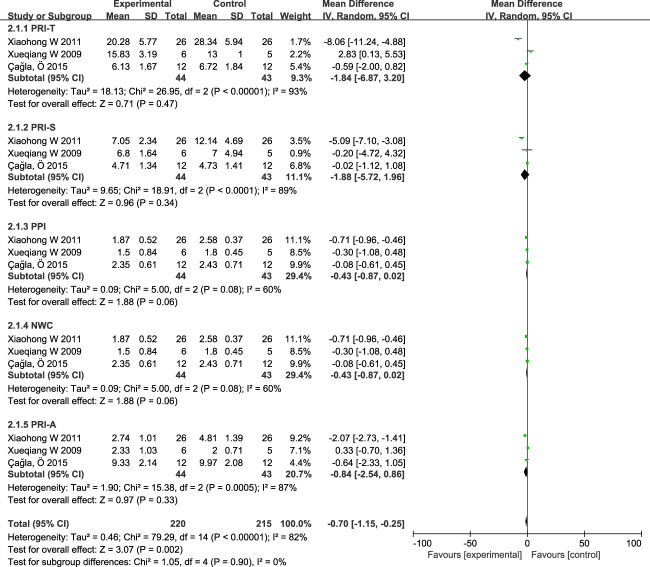
Forest plot of SF-MPQ. Compared with control groups, SF-MPQ scores in TENS groups were decreased. (‘‘mean’’ represent the mean of VAS for each study, ‘‘total’’ represents how many patients were involved in the study, ‘‘weighted’’ represents the weight of each study in the meta-analysis).

There were three studies [24, 26, 27] using SF-MPQ as an outcome indicator, with a total of 87 individuals included for comparison. The results of the heterogeneity analysis showed that: I^2^ = 82%, *P* < 0.0001, there was large heterogeneity between study results and the random effect model was being used, as shown in Fig. 3. The results showed that the SF-MPQ scores in the TENS groups were generally lower than those in the control groups (the mean difference was −0.70, 95%CI, −1.15 to −0.25, *P* = 0.002). And it is considered that compared to the control group, the scores in the SF-MPQ of people with pain after spinal cord injury were also reduced after transcutaneous electrical nerve stimulation treatment.

### Sensitivity analysis results

Sensitivity analysis was performed by STATA 12.0 software. Sensitivity analysis for the SF-MPQ revealed that the heterogeneity across subgroups changed after excluding one study, Xia B [24], suggests that this study was the source of the heterogeneity in this result. The results are shown in supplementary figure 3.

### Publishing bias analysis

Using STATA 12.0 software, we used funnel plots to compare effect sizes to assess publication bias and selected MD as the effect size. The results are shown in Supplementary Fig. 4. Due to the small number of literature included in this study and the limited usefulness of the funnel plot, the Egger test was used to test for publication bias in the quantitative study. The results showed: *P* = 0.303, no publication bias. The results are shown in Supplementary Fig. 5.

## Discussion

The spinal cord is an essential site as this is where different incoming sensory and nociceptive signals undergo convergence and modulation [28]. Post-SCI pain is a significant symptom of SCI. Pain can have a specific psychological impact, interfere with sleep, make patients uncooperative with rehabilitation exercises, and affect functional recovery, thus thereby significantly reducing the quality of life. Musculoskeletal pain is most commonly observed after SCI and is associated with peripheral pain mechanisms, with primary afferent nociceptor activation and signal transmission to the brain along pain pathways. Visceral pain can be caused by pain caused by disease, inflammation, or swelling of visceral structures. The afferent nerves of the vagus, visceral, and pelvic nerves innervate the thoracic, abdominal, and pelvic organs and project to different ganglia, dorsal horns, and central neurons [29]. The mechanism of neuropathic pain is more complex and may be correlated with changes in the surrounding environment, spinal cord, and brain. In the physiological state, activation of injured unmyelinated (C-Fiber) and sparsely myelinated (Aδ-fiber) afferent fibers indicates tissue damage, which is reflected by high thresholds of nociceptive receptors for mechanical, thermal, and chemical stimuli. These thresholds vary significantly with pain [30, 31]. After SCI, sodium channels generated by neurons in the dorsal horn of the spinal cord can be altered, allowing neurons to respond to unaltered peripheral stimuli at a rate higher than normal and produce spontaneous misfires in the absence of stimuli, resulting in pain [32]. Changes in the thalamocortex and other subcortical structures after SCI indicate that pain plays a role in the brain after SCI.

TENS is a type of electrotherapy primarily relieves pain clinically. The current study aimed to assess whether analgesic effects are achieved via different neurobiological mechanisms affecting the peripheral and central nervous systems [9]. A previous study investigating pain in mice with pain disorders has shown that TENS relieves nociceptive hyperalgesia and maintains spinal opioid receptors by inhibiting mitogen-activated protein kinase and proinflammatory cytokine expression to activate PKC-γ and P-CREB [33]. Moreover, Pfyffer et al. [34] have revealed that TENS can excite crude fibers and activate glial cells to release inhibitory neurotransmitters. Thus, the injury sensory signals generated from the same segment of fine fibers on the spinal dorsal horn of the projected neuron’s excitatory action, are inhibited. In addition, the gate of pain is closed and uploaded to the center of the injury nerve impulse reduction, thereby facilitating pain relief. Simultaneously, the central nervous system can release endogenous analgesic substances and activate endogenous analgesia system receptors, and can have a thermal effect, which reduces the excitability of the sensory nerve. Another study has shown a reduction in dorsal horn neuron activity in control animals and primary and secondary hyperalgesia in animals with acute inflammation following TENS treatment [35]. Therefore, it has a special therapeutic effect on both central and peripheral pain, and is commonly used in clinical practice; however, the therapeutic efficacy of TENS against pain after SCI remains controversial.

## Summary of the results of the study

This study assessed six articles—two in Chinese and four in English—all of which used the VAS score as an indicator of pain relief, and three utilized the SF-MPQ for the comprehensive assessment of pain improvement. Based on the literature quality assessment results, the quality of the articles was moderate; therefore, the results of the analysis are moderately accurate. The meta-analysis results showed that the VAS and SF-MPQ scores of the treatment group decreased compared with those of the control group. Therefore, TENS can improve pain symptoms among people with SCI. However, only one study conducted by Xia B [24] had a follow-up. Thus, the long-term assessments of pain in persons with SCI treated with TENS are commonly insufficient, and there is a lack of evaluation and follow-up of long-term efficacy and quality of life at a later stage.

## Limitations and heterogeneous analysis

This had meta-analysis several limitations. First, as the number of studies in this field is low, only six studies were analyzed. Some randomization grouping methods were not clearly stated, blinding was not specified in all studies. Further, random assignment concealment was rarely reported. At the outcome level, all studies used the VAS scores to assess pain, and only three studies utilized the SF-MPQ to comprehensively evaluate pain. In addition, the efficacy index was not uniform. Only one of the six studies described long-term follow-up. Hence, the duration of pain relief, long-term quality of survival, and presence of adverse effects were not evaluated, thereby indicating a lack of studies about long-term efficacy. The studies did not classify pain after SCI. Therefore, we could not identify differences in TENS treatment in the varying types of SCI and further studies must be conducted to assess these aspects.

A large heterogeneity was observed in the indicator scores in the SF-MPQ. If Xia B’s study^24^ was excluded, the reversal of the heterogeneity of the PRI-S and PRI-A results showed that the study was a source of heterogeneity. The source of heterogeneity in the study may be as follows: Electrodes in TENS are commonly placed on both sides of the painful area, and a small number of studies placed the electrodes on both sides of the spine, in opposing directions, or in juxtaposition. The pulse frequency of TENS differed. The maximum pulse frequency was 100 Hz, and the minimum pulse frequency was only 2 Hz. The treatment time varied from 30 min per day in some studies to 20 min per day in others, and the duration differed from 10 days to 12 weeks.

## Clinical suitability and outlook

There is currently no definitive treatment for pain in people with SCI. However, TENS can improve blood circulation at the affected site and improve pain and is now widely used in clinical practice. The current study aimed to validate whether TENS can significantly improve pain symptoms in SCI. Results showed that it could be used alone or in combination with other modalities to improve pain symptoms among individuals with SCI. However, its long-term efficacy should be further evaluated.

## Conclusion

TENS could relieve pain in persons with SCI. However, only a few studies were included. Hence, standardized randomized controlled trials with larger sample sizes and long-term follow-up must be conducted to provide more reliable results.

## Supplementary information

Supplementary Figure 1

Supplementary Figure 2

Supplementary Figure 3

Supplementary Figure 4

Supplementary Figure 5

## Data Availability

The datasets generated and/or analyzed during the current study are available from the major public websites.
