# Supplementary material for: Efficacy of transcutaneous electrical nerve stimulation in people with pain after spinal cord injury: a meta-analysis
Source: Spinal Cord. 2022 Mar 11;60(5):375–81. doi: 10.1038/s41393-022-00776-z (PMC9106573; doi:10.1038/s41393-022-00776-z)
Supplement: Supplementary file 1 — Supplementary Figure 1 [file 41393_2022_776_MOESM1_ESM.pdf]

#1. "Spinal Cord Injuries"[Mesh]

#2. ((((((Spinal Cord Trauma*[Title/Abstract]) OR (Traumatic Myelopath*[Title/Abstract])) OR (Spinal Cord Injur*[Title/Abstract])) OR (Spinal CordTransection*[Title/Abstract])) OR (Spinal Cord Laceration*[Title/Abstract])) OR (Post Traumatic Myelopath*[Title/Abstract])) OR (Spinal Cord Contusion*[Title/Abstract])

#3. #1 OR #2

#4. "Pain"[Mesh]

#5. ((pain[Title/Abstract]) OR (ache*[Title/Abstract]))

#6. #4 OR #5

#7. "Transcutaneous Electric Nerve Stimulation"[Mesh]

#8. (((((((((((Transcutaneous Electric Stimulation[Title/Abstract]) OR (Percutaneous Electric Nerve Stimulation[Title/Abstract])) OR (TENS[Title/Abstract])) OR (Transcutaneous Electrical Stimulation[Title/Abstract])) OR (Transdermal Electrostimulation[Title/Abstract])) OR (Percutaneous Electrical Nerve Stimulation[Title/Abstract])) OR (Transcutaneous Electrical Nerve Stimulation[Title/Abstract])) OR (Transcutaneous Nerve Stimulation[Title/Abstract])) OR (Percutaneous Neuromodulation Therap*[Title/Abstract])) OR (Percutaneous Electrical Neuromodulation*[Title/Abstract])) OR (Analgesic Cutaneous Electrostimulation[Title/Abstract])) OR (Electroanalgesia*[Title/Abstract])

#9. #7 OR #8

#10. #3 AND #6 AND #9

#11. randomized controlled trial[Publication Type] OR (randomized[Title/Abstract] AND controlled[Title/Abstract] AND trial[Title/Abstract])

#12. #10 AND #11
