# Supplementary figures and images for: Efficacy of transcutaneous electrical nerve stimulation in people with pain after spinal cord injury: a meta-analysis
Source: Spinal Cord. 2022 Mar 11;60(5):375–81. doi: 10.1038/s41393-022-00776-z (PMC9106573; doi:10.1038/s41393-022-00776-z)

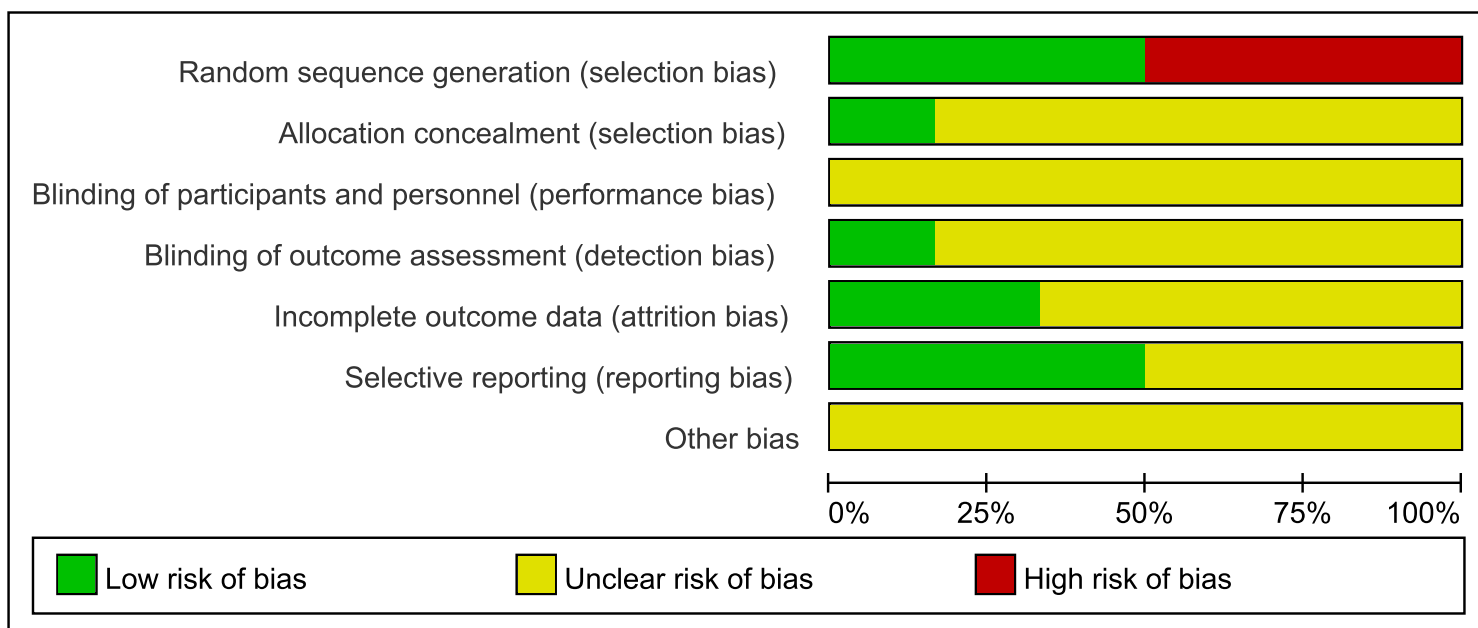

Supplement: Supplementary file 2 — Supplementary Figure 2 [file 41393_2022_776_MOESM2_ESM.pdf]
